# Correlation between early cumulative fluid balance and 90-day all-cause mortality in patients with acute pancreatitis: a retrospective cohort study

**DOI:** 10.1016/j.clinsp.2026.101019

**Published:** 2026-06-02

**Authors:** Jianlei Chen, Jinyuan Chi, Hao Li

**Affiliations:** Department of Hepatobiliary and Pancreatic Surgery, Yanbian University Hospital, Yanbian, China

**Keywords:** Acute pancreatitis, Fluid balance, Positive balance, Negative balance, 90-day all-cause mortality

## Abstract

**Objective:**

Aggressive early hydration is the cornerstone of Acute Pancreatitis (AP) care, but the optimal fluid-balance strategy and its impact on long-term survival remain unclear. The authors therefore tested whether first-week fluid accumulation affects 90-day mortality.

**Methods:**

Retrospective cohort study of 1142 AP patients admitted to the Intensive Care Unit (ICU) within the MIMIC-IV database. Daily fluid intake/output were extracted for the first 7-days; patients were classified as cumulative positive (*n* = 842) or negative (*n* = 300) balance by Day-7. The authors used Cox regression, Propensity Score Matching (PSM), and multiple sensitivity analyses to evaluate the association between fluid balance and 90-day mortality.

**Results:**

Overall 90-day mortality was 19.3%. Mortality was higher in the positive balance group. Multivariable Cox regression showed an adjusted hazard ratio (HR = 1.54 [1.06–2.22], *p* = 0.022); the corresponding E-value was 2.45. After PSM, the association persisted. IPTW yielded a similar point estimate (HR = 1.51, 95% CI 1.01–2.25, *p* = 0.115). Landmark analyses excluding early deaths confirmed the finding. RCS revealed a non-linear relationship, suggesting both highly positive and negative balances are associated with increased mortality.

**Conclusion:**

A positive cumulative fluid balance at Day-7 is associated with increased 90‑day mortality in ICU patients with AP. This association may partially reflect the higher severity of the underlying disease process in the group with positive cumulative fluid balance on Day-7, rather than a direct causal effect. Despite this limitation, the present findings support an individualized, biphasic fluid strategy: early adequate resuscitation followed by goal‑directed de‑escalation.

## Highlights

• Positive day-7 fluid balance linked to higher 90-day mortality in acute pancreatitis ICU patients.
• Nonlinear U-shaped relation: both high positive and negative balances increase risk.
• Biphasic fluid strategy suggested: early resuscitation, then goal-directed de-escalation.
• First large-scale MIMIC-IV study quantifying 7-day fluid balance impact on AP survival.

## Introduction

Acute Pancreatitis (AP) constitutes a leading gastrointestinal indication for acute hospital admissions internationally, with population-based studies reporting an annual incidence rate of 30–40 cases per 100,000 inhabitants. This incidence continues to rise in both Western and Asian countries.^1^ The pathogenesis primarily centers on premature activation of pancreatic enzymes, leading to acinar cell injury, sterile inflammation, and pancreatic microcirculatory failure, which can rapidly extend to extrapancreatic organs.^2^ Most AP patients experience a self-limiting course; however, approximately 20% progress to Severe Acute Pancreatitis (SAP), developing organ failure and/or local complications such as pancreatic necrosis, pseudocysts, or encapsulated necrosis. Should SAP progress to infected pancreatic necrosis concomitant with Multiple-Organ-Dysfunction Syndrome (MODS), case-fatality estimates converge on approximately one in three patients.^3^

Fluid therapy plays a critical role in managing critically ill patients, particularly those with early-stage AP.^4^ Fluid resuscitation is a core early intervention for AP, rapidly correcting hypovolemia, maintaining microcirculatory perfusion, and mitigating progressive ischemia-reperfusion injury to the pancreas and distant organs. This approach reduces the risk of Systemic Inflammatory Response Syndrome (SIRS) and persistent organ dysfunction.^5^

Previous studies have explored the efficacy of early aggressive fluid resuscitation in reducing mortality risk among AP patients.^6^ However, existing evidence remains limited regarding the relationship between fluid input, fluid balance, and diuresis. Excessive fluid resuscitation not only fails to improve microcirculation but may also lead to Abdominal Compartment Syndrome (ACS), Acute Respiratory Distress Syndrome (ARDS), and renal overload.^7^, ^8^, ^9^ An observational study further confirmed that massive fluid loading and cumulative positive fluid balance adversely affect survival outcomes in critically ill patients.^10^ Previous findings indicate that mortality rates in critically ill patients with acute kidney injury, sepsis, and acute lung injury are closely associated with fluid overload.^11^, ^12^, ^13^ In recent years, some researchers have challenged the early aggressive intravenous fluid therapy approach for AP. Their studies suggest that early aggressive fluid resuscitation does not significantly improve mortality rates and may even increase the risk of renal injury and pulmonary edema, potentially leading to renal insufficiency and respiratory failure.^14^ Consequently, a therapeutic equilibrium that secures adequate intravascular volume without precipitating fluid accumulation remains a pivotal ‒ and still unresolved ‒challenge in the early care of AP, especially in its severe phenotype, and warrants further in-depth exploration.

To date, specific data on fluid management in AP patients remains unavailable. Consequently, the team conducted a retrospective study of Intensive Care Unit (ICU) patients from an open-source database, performing a detailed analysis of early daily fluid dynamics. The main objective was to delineate how cumulative fluid status modulates clinical outcomes in individuals with AP.

## Materials and methods

### Database overview

The data for this study were sourced from the Medical Information Mart for Intensive Care-IV v3.1 (MIMIC-IV v3.1) database. This is a large, publicly accessible database (https://physionet.org/content/mimiciv/3.1/https://physionet.org/content/mimiciv/3.1/) encompassing approximately 500,000 inpatient cases from Beth Israel Deaconess Medical Center (BIDMC) between 2008 and 2022.^15^ To access this database, the study's lead author, Jianlei Chen, successfully completed the CITI training program and passed the “Conflict of Interest” and “Data or Specimen Only Research” examinations (ID: 67,992,428). Given that all data in this repository is de-identified and permits research resource sharing, the BIDMC Institutional Review Board (IRB) waived the informed consent requirement. The BIDMC IRB approval number is 2001-P-001,699/14, and the Massachusetts Institute of Technology (MIT) IRB approval number is 0403,000,206. All reports follow the guidelines for reporting observational studies in epidemiology (STROBE).

### Study population selection criteria

**Fig. 1:**
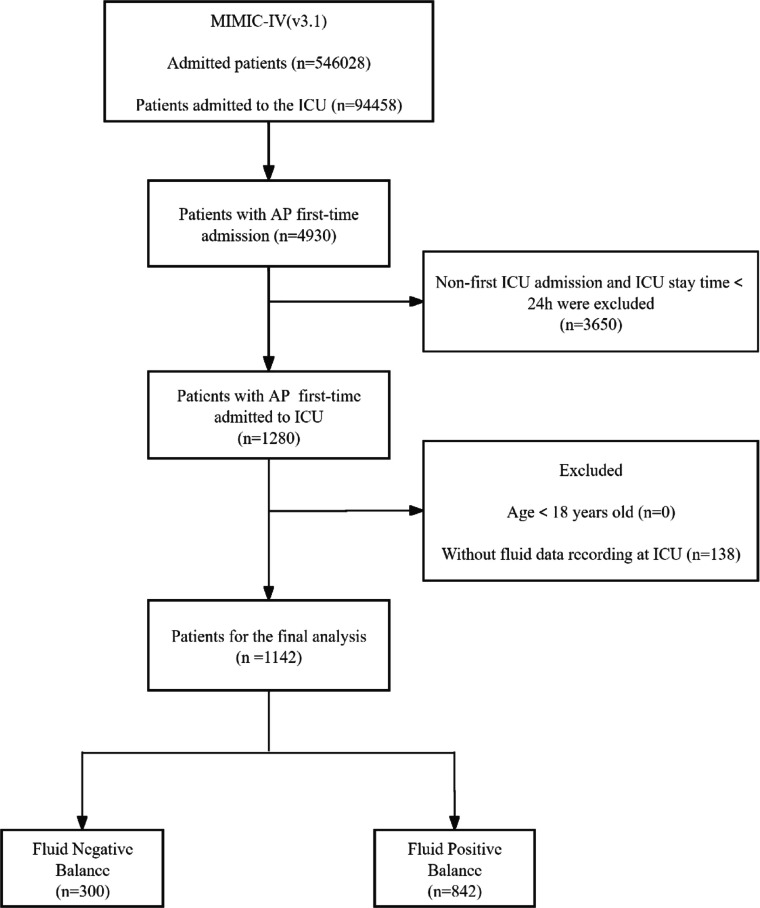
The flowchart details the inclusion and exclusion criteria, ultimately showing that 1142 study subjects had data available for final analysis.

A total of 4930 AP patients were extracted based on International Classification of Diseases, 9th Revision (ICD-9) code 577.0 and 10th Revision (ICD-10) codes K85-K85.92. Among these, 1280 AP patients were admitted to the ICU. Following rigorous inclusion and exclusion criteria (Fig. 1), patients meeting the following criteria were excluded: 1) Subjects younger than 18-years; 2) Among individuals with more than one ICU stay, only data from the first admission were retained; 3) ICU stay <24 h; 4) Absence of fluid administration records after ICU admission. Ultimately, 1142 AP patients were included.

### Clinical and laboratory data extraction

All data were retrieved through structured queries executed in PostgreSQL (version 17.3). Baseline information was collected during the first assessment after ICU admission, encompassing the following general data: Demographics (age, gender, race), vital signs (heart rate, systolic blood pressure, diastolic blood pressure, mean arterial pressure, respiratory rate, oxygen saturation, body temperature), clinical interventions (use of dopamine, epinephrine, norepinephrine, octreotide, mechanical ventilation, and continuous renal replacement therapy), comorbidities (Congestive Heart Failure [CHF], Chronic Obstructive Pulmonary Disease [COPD], diabetes, kidney disease, malignancy, severe liver disease, sepsis), and Sequential Organ Failure Assessment (SOFA) score, Acute Physiology Score III (APS-III), and Charlson Comorbidity Index.

The study did not differentiate fluid types (such as crystalloid or colloid) and extracted daily fluid intake for the first 7-days. Cumulative fluid balance was defined as the difference between cumulative fluid input (including intravenous drugs, bolus fluids, blood components, enteral or parenteral nutrition, and standing maintenance infusions) and cumulative fluid output (including urine output, dialysis, and postoperative drainage). Positive fluid balance was defined as a positive cumulative fluid balance (> 0mL) on Day-7 of hospitalization, at discharge, or at death. Negative cumulative fluid balance was defined as a negative cumulative fluid balance (< 0mL). Patients were classified as positive or negative based on the sign of their cumulative fluid balance value.^16^

### Primary study variables and outcomes

Cumulative fluid balance over the first seven days served as the principal exposure, with all-cause mortality within 90-days of ICU entry designated as the primary endpoint.

### Statistical analysis

Patients were categorized into positive balance and negative balance groups based on cumulative fluid balance. Categorical variables were expressed as proportions (%) and tested using Chi-Square or Fisher's exact tests. Normally distributed continuous variables were presented as mean ± Standard Deviation (SD) and compared using Student's *t*-tests or one-way analysis of variance (ANOVA). For continuous variables not meeting normality assumptions, data were presented as median and Interquartile Range (IQR) and analyzed using the Kolmogorov-Smirnov test. For covariates with missing values <10%, the mean or median of the variable was used as a proxy.

Identify potential risk factors through univariate Cox regression analysis, and incorporate covariates with p-values < 0.05 into the multivariate. The authors employed Cox regression analysis to quantify the relationship between fluid-balance status and the risk of death within 90-days. Crude survival probabilities were depicted with Kaplan-Meier (K-M) curves and compared by the log-rank test.

Additional analyses were performed to strengthen the present findings: Effect consistency was further explored through prespecified subgroup analyses and interaction testing across clinically relevant strata.

To account for potential bias, Propensity Score Matching (PSM) was employed to identify variables associated with fluid balance. A 1:1 nearest neighbor matching algorithm with a caliper width of 0.25 was used. Post-matching, univariate and multivariate Cox models together with Kaplan-Meier survival were fitted to quantify the isolated effect of fluid balance on 90-day mortality after matching. Balance diagnostics relied on Standardized Mean Differences (SMDs) to verify adequate covariate equalization.^17^

To assess the robustness of these primary findings to potential unmeasured confounding, the authors calculated the E-value for the hazard ratio of fluid balance on 90-day mortality.^18^ Recognizing the limitations of propensity score matching in controlling for unmeasured confounders, the authors employed Inverse Probability of Treatment Weighting (IPTW) to further evaluate the effect of 7-day cumulative fluid balance on 90-day mortality.

To address potential immortal time bias, the authors conducted a landmark analysis. By excluding patients who died within the first 24-, 48-, or 72 h, the authors compared survival rates across fluid balance status, conditional on survival up to 90-days.

The nonlinear association between 7-day cumulative fluid balance and mortality risk was explored using Restricted Cubic Spline (RCS) models.

All statistical analyses were performed using R software version 4.2.2 (http://www.R-project.org, The R Foundation) and Free Statistics software version 2.1.1. A two-tailed *p* < 0.05 was set as the threshold for statistical significance.

## Results

### Baseline characteristics of the study population

Table 1 presents baseline characteristics of the study population. A total of 1142 patients were enrolled. The 90-day mortality rate across the entire cohort was 19.3%, with rates of 12.7% in the negative balance group and 21.6% in the positive balance group. Roughly half of the patients were aged 60 years or older, predominantly Caucasian, with 58.0% males and 42.0% females. Notably, the positive balance group exhibited higher APS-III, SOFA scores, and greater utilization of life-sustaining therapies (epinephrine, norepinephrine, and CRRT), indicating greater baseline illness severity in this cohort (*p* < 0.05). All remaining variables showed comparable distributions between cohorts.

**Table 1:** Baseline characteristics of participants.

Variables	Total (*n* = 1142)	Negative balance (*n* = 300)	Positive balance (*n* = 842)	p
Gender, n (%)				0.282
Female	480 (42.0)	134 (44.7)	346 (41.1)	
Male	662 (58.0)	166 (55.3)	496 (58.9)	
Age, n (%)				0.105
< 60	586 (51.3)	166 (55.3)	420 (49.9)	
≥ 60	556 (48.7)	134 (44.7)	422 (50.1)	
Race, n (%)				0.332
White	718 (62.9)	187 (62.3)	531 (63.1)	
Black	124 (10.9)	27 (9)	97 (11.5)	
Others	300 (26.3)	86 (28.7)	214 (25.4)	
HR, Mean ± SD	99.3 ± 22.1	97.5 ± 22.3	100.0 ± 22.0	0.1
SBP, Mean ± SD	127.8 ± 25.8	129.7 ± 25.5	127.2 ± 26.0	0.145
DBP, Mean ± SD	73.8 ± 19.2	73.7 ± 18.5	73.8 ± 19.5	0.982
MAP, Mean ± SD	87.1 ± 20.2	87.7 ± 20.7	86.9 ± 20.0	0.577
RR, Mean ± SD	21.4 ± 6.6	21.3 ± 6.7	21.4 ± 6.6	0.911
Temperature, Mean ± SD	36.8 ± 0.9	37.0 ± 0.8	36.8 ± 1.0	<0.001
SpO_2_, Mean ± SD	96.0 ± 4.1	96.2 ± 4.2	96.0 ± 4.0	0.586
CHF, n (%)				0.013
No	913 (79.9)	225 (75)	688 (81.7)	
Yes	229 (20.1)	75 (25)	154 (18.3)	
COPD, n (%)				<0.001
No	907 (79.4)	217 (72.3)	690 (81.9)	
Yes	235 (20.6)	83 (27.7)	152 (18.1)	
Diabetes, n (%)				0.189
No	780 (68.3)	214 (71.3)	566 (67.2)	
Yes	362 (31.7)	86 (28.7)	276 (32.8)	
Renal disease, n (%)				0.086
No	929 (81.3)	254 (84.7)	675 (80.2)	
Yes	213 (18.7)	46 (15.3)	167 (19.8)	
Cancer, n (%)				0.364
No	1053 (92.2)	273 (91)	780 (92.6)	
Yes	89 (7.8)	27 (9)	62 (7.4)	
Liver disease, n (%)				0.472
No	1015 (88.9)	270 (90)	745 (88.5)	
Yes	127 (11.1)	30 (10)	97 (11.5)	
Sepsis, n (%)				0.391
No	437 (38.3)	121 (40.3)	316 (37.5)	
Yes	705 (61.7)	179 (59.7)	526 (62.5)	
Charlson average score, Median (IQR)	4.0 (2.0, 6.0)	3.0 (1.0, 6.0)	4.0 (2.0, 6.0)	0.084
APSIII average score, Mean ± SD	52.1 ± 24.3	45.7 ± 20.3	54.4 ± 25.1	<0.001
SOFA average score, Median (IQR)	3.8 (1.7, 6.6)	3.1 (1.7, 5.7)	4.0 (1.8, 6.9)	0.001
Epinephrine, n (%)				0.013
No	1090 (95.4)	294 (98)	796 (94.5)	
Yes	52 (4.6)	6 (2)	46 (5.5)	
Dopamine, n (%)				0.306
No	1114 (97.5)	295 (98.3)	819 (97.3)	
Yes	28 (2.5)	5 (1.7)	23 (2.7)	
Norepinephrine, n (%)				0.029
No	808 (70.8)	227 (75.7)	581 (69)	
Yes	334 (29.2)	73 (24.3)	261 (31)	
Octreotide, n (%)				0.116
No	1072 (93.9)	276 (92)	796 (94.5)	
Yes	70 (6.1)	24 (8)	46 (5.5)	
CRRT, n (%)				<0.001
No	1004 (87.9)	288 (96)	716 (85)	
Yes	138 (12.1)	12 (4)	126 (15)	
Ventilator, n (%)				0.273
No	270 (23.6)	64 (21.3)	206 (24.5)	
Yes	872 (76.4)	236 (78.7)	636 (75.5)	
90-d mortality, n (%)				<0.001
No	922 (80.7)	262 (87.3)	660 (78.4)	
Yes	220 (19.3)	38 (12.7)	182 (21.6)	

### Cumulative fluid input, balance volume, and diuresis

**Fig. 2:**
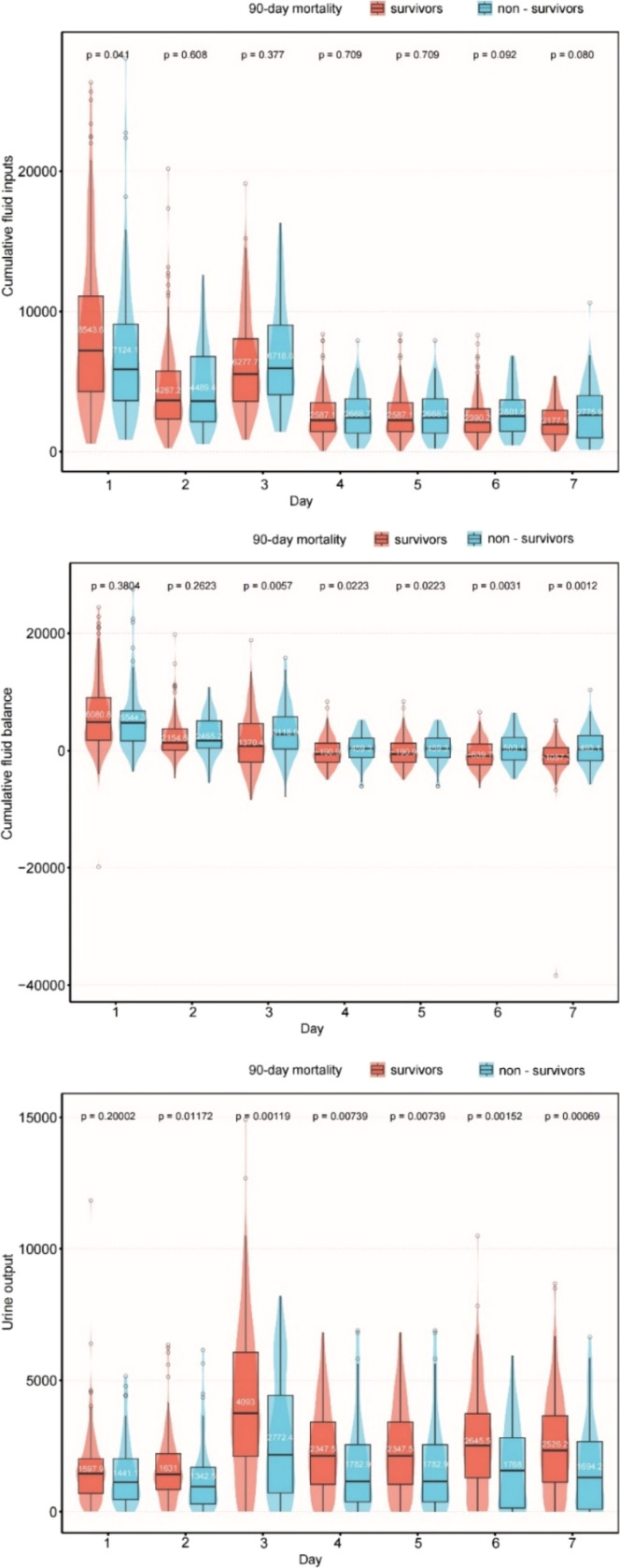
Fluid intake, diuresis, and fluid balance in the 90-day survival and mortality groups during the first 7-days.

Fig. 2 illustrates the daily fluid dynamics over the first seven days. It shows the cumulative daily fluid intake, fluid balance, and diuresis for both the survivor and non-survivor groups from Day-1 to Day-7. On Day-1, fluid intake was significantly higher in survivors than in non-survivors, suggesting that early adequate resuscitation may be beneficial. However, from Day-3 onward, survivors achieved a more negative cumulative fluid balance, whereas non-survivors remained in persistent positive balance. Notably, from Day-2 to Day-7, urine output was consistently lower in non-survivors, indicating impaired renal function in this group. These findings highlight the dynamic nature of fluid balance: survivors were characterized by high initial intake followed by gradual fluid removal, whereas non-survivors were marked by sustained positive balance and oliguria.

### Relationship between fluid balance and 90-day survival

**Fig. 3:**
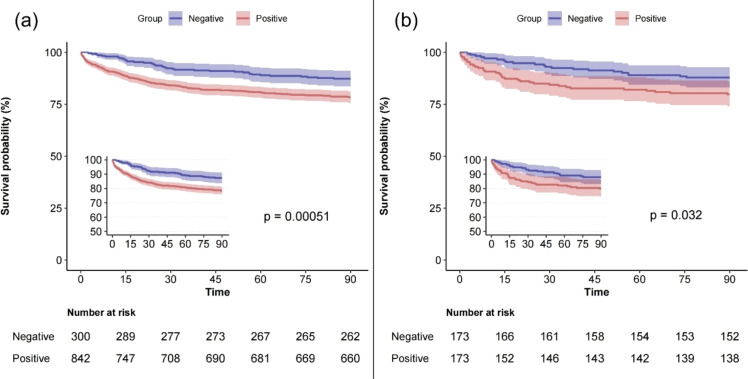
Kaplan-Meier survival curves for cumulative 7-day fluid balance and 90-day mortality. (a) Pre-PSM K-M survival analysis; (b) Post-PSM K-M survival analysis.

Table 2 presents univariate and multivariate Cox regression analyses. In the univariate Cox regression analysis, positive fluid balance was significantly associated with 90-day mortality (Hazard Ratio [HR] = 1.84 [1.3–2.61], *p* < 0.001). After adjusting for relevant covariates, positive fluid balance remained independently associated with mortality in the Cox multivariate regression analysis (HR = 1.54 [1.06–2.22], *p* = 0.022), this translates to an approximate doubling of the risk of death. The K-M survival curves in Fig. 3a, grouped by fluid balance, analyzed the relationship with 90-day mortality risk. Results showed that the 90-day mortality risk was significantly higher in the positive fluid balance group compared to the negative fluid balance group.

**Table 2:** Univariate and multivariate Cox regression analysis of fluid balance and 90-day mortality.

Variables	Univariate analysis		Multivariate analysis	
	HR (95% CI)	p	HR (95% CI)	p
Male, n (%)	0.95 (0.73, 1.24)	0.71		
Age ≥ 60, n (%)	2.43 (1.83, 3.22)	<0.001	1.51 (1.06∼2.15)	0.022
Race, n (%)				
White	Ref			
Black	0.73 (0.44, 1.19)	0.204		
Others	1.15 (0.86, 1.55)	0.34		
HR, Mean ± SD	0.9989 (0.9929, 1.0049)	0.717		
SBP, Mean ± SD	0.99 (0.98, 0.99)	<0.001	1 (0.99∼1.01)	0.644
DBP, Mean ± SD	0.98 (0.98, 0.99)	<0.001	0.99 (0.97∼1.01)	0.269
MAP, Mean ± SD	0.99 (0.98, 0.99)	<0.001	1.01 (0.99∼1.03)	0.468
RR, Mean ± SD	1.03 (1.01, 1.05)	0.006	1.02 (1∼1.04)	0.049
Temperature, Mean ± SD	0.74 (0.68, 0.82)	<0.001	0.94 (0.84∼1.05)	0.294
SpO_2_, Mean ± SD	0.96 (0.93, 0.98)	0.003	0.99 (0.96∼1.02)	0.501
CHF, n (%)	1.63 (1.21, 2.19)	0.001	0.98 (0.69∼1.39)	0.917
COPD, n (%)	1.19 (0.87, 1.63)	0.271		
Diabetes, n (%)	1.0082 (0.7591, 1.3389)	0.955		
Renal disease, n (%)	2.21 (1.67, 2.94)	<0.001	0.75 (0.51∼1.08)	0.124
Cancer, n (%)	3.11 (2.21, 4.38)	<0.001	1.54 (0.97∼2.45)	0.064
Liver disease, n (%)	2.57 (1.87, 3.54)	<0.001	1.51 (0.98∼2.31)	0.06
Sepsis, n (%)	2.49 (1.81, 3.44)	<0.001	0.93 (0.64∼1.36)	0.719
Charlson average score, Median (IQR)	1.25 (1.21, 1.3)	<0.001	1.19 (1.11∼1.28)	<0.001
APSIII average score, Mean ± SD	1.03 (1.03, 1.04)	<0.001	1.02 (1.01∼1.03)	<0.001
SOFA average score, Median (IQR)	1.2 (1.17, 1.24)	<0.001	1 (0.94∼1.06)	0.948
Epinephrine, n (%)	6.38 (4.42, 9.21)	<0.001	3.51 (2.28∼5.42)	<0.001
Dopamine, n (%)	3.32 (1.93, 5.7)	<0.001	1.81 (1.02∼3.21)	0.043
Norepinephrine, n (%)	4.07 (3.12, 5.33)	<0.001	1.74 (1.19∼2.54)	0.004
Octreotide, n (%)	2.01 (1.3, 3.09)	0.002	1.54 (0.94∼2.55)	0.089
CRRT, n (%)	4.44 (3.34, 5.9)	<0.001	0.88 (0.58∼1.31)	0.52
Ventilator, n (%)	1.6 (1.12, 2.28)	0.009	0.73 (0.49∼1.08)	0.119
Cumulative fluid inputs, Mean ± SD	1 (1,1)	<0.001		
Cumulative fluid outputs, Mean ± SD	1 (1,1)	0.793		
Cumulative fluid balance, Mean ± SD	1 (1,1)	<0.001		
Urineoutput, Mean ± SD	1 (0.9999, 1)	0.001		
Cumulative fluid positive balance, n (%)	1.84 (1.3, 2.61)	<0.001	1.54 (1.06∼2.22)	0.022

### Subgroup analysis

**Fig. 4:**
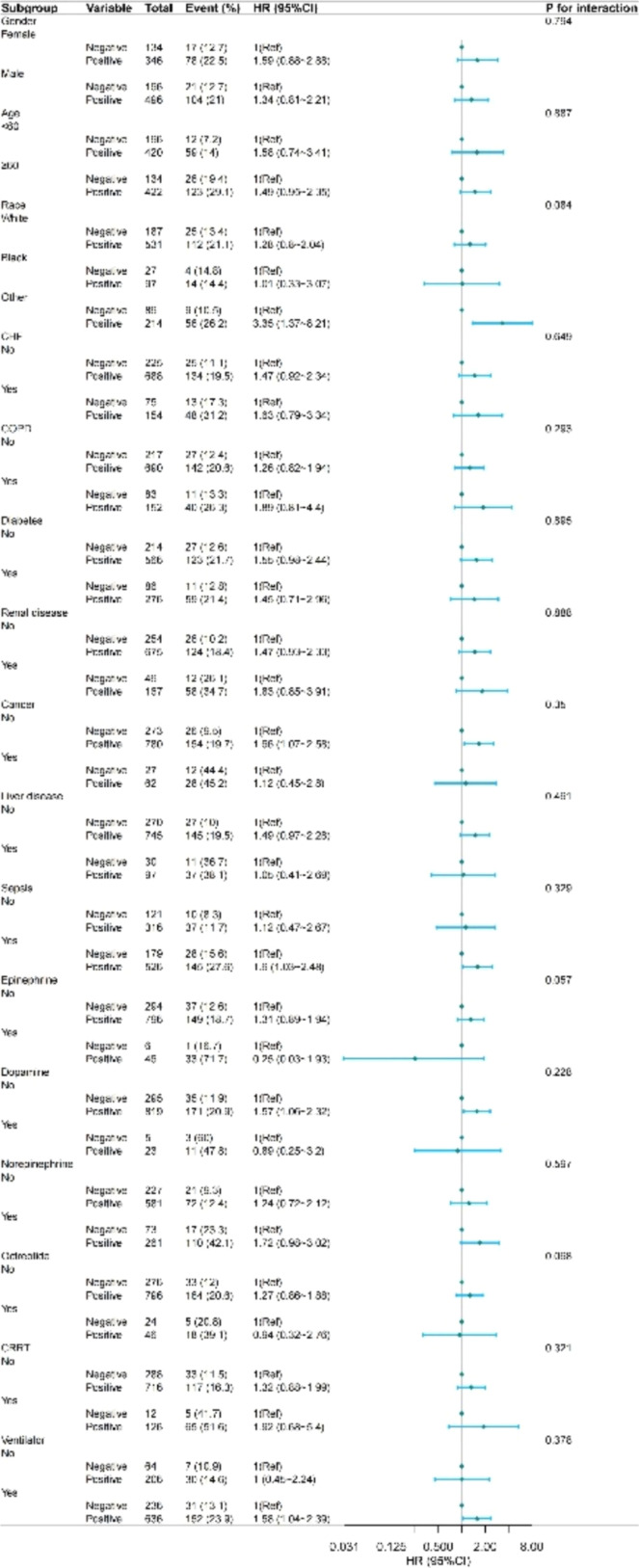
Subgroup analysis forest plot of fluid balance and 90-day mortality.

The authors performed subgroup analyses to evaluate the consistency of the association between positive fluid balance and 90-day mortality across different patient characteristics (Fig. 4). Tests for interaction revealed no statistically significant effect modification for most variables (*p* > 0.05). Although the direction of the effect was consistent across most subgroups, the confidence intervals were wide for many subgroups, reflecting limited sample sizes and imprecise estimates, thus precluding definitive conclusions regarding effect modification. Overall, these subgroup analyses were exploratory but lacked sufficient statistical power to detect heterogeneity.

### Sensitivity analysis and landmark analysis

Post-PSM comparisons of matched pairs and SMDs are detailed in Supplementary File 1 to Supplementary File 3. Patients were divided into positive and negative fluid balance groups based on fluid balance, with 173 matched pairs per group achieving covariate balance. Cox univariate and multivariate analyses of PSM-matched patients revealed that positive fluid balance remained independently associated with 90-day mortality (HR = 2.17 [1.18–4], *p* = 0.013) (Table 3). In the PSM dataset, K-M survival analysis still revealed a persistently higher mortality risk for patients with positive fluid balance. (Fig. 3b).

**Table 3:** Univariate and multivariate Cox regression analysis of post-PSM fluid balance and 90-day mortality.

Variables	Univariate analysis		Multivariate analysis	
	HR (95% CI)	p	HR (95% CI)	p
Male, n (%)	0.83 (0.49, 1.4)	0.487		
Age ≥ 60, n (%)	2.33 (1.34, 4.05)	0.003	1.41 (0.62∼3.2)	0.406
Race, n (%)				
White	Ref			
Black	0.97 (0.41, 2.31)	0.942		
Others	0.86 (0.48, 1.56)	0.622		
HR, Mean ± SD	0.9931 (0.9802, 1.0061)	0.296		
SBP, Mean ± SD	0.99 (0.98, 1)	0.024	1.01 (0.99∼1.02)	0.389
DBP, Mean ± SD	0.99 (0.97, 1)	0.103		
MAP, Mean ± SD	0.9915 (0.9778, 1.0054)	0.229		
RR, Mean ± SD	1.0084 (0.9674, 1.0511)	0.694		
Temperature, Mean ± SD	0.55 (0.45, 0.67)	<0.001	0.64 (0.48∼0.84)	0.001
SpO_2_, Mean ± SD	0.97 (0.92, 1.01)	0.157		
CHF, n (%)	1.5 (0.87,2.58)	0.142		
COPD, n (%)	1.08 (0.6, 1.92)	0.806		
Diabetes, n (%)	1.03 (0.58, 1.83)	0.911		
Renal disease, n (%)	2.04 (1.15, 3.6)	0.014	0.81 (0.39∼1.7)	0.579
Cancer, n (%)	3.59 (1.96, 6.57)	<0.001	1.99 (0.85∼4.66)	0.113
Liver disease, n (%)	2.84 (1.57, 5.12)	<0.001	1.96 (0.81∼4.73)	0.133
Sepsis, n (%)	1.04 (0.55,1.96)	0.916		
Charlson average score, Median (IQR)	1.25 (1.16, 1.33)	<0.001	1.21 (1.06∼1.39)	0.006
APSIII average score, Mean ± SD	1.03 (1.03, 1.04)	<0.001	1.02 (1∼1.03)	0.033
SOFA average score, Median (IQR)	1.27 (1.18, 1.37)	<0.001	1.04 (0.9∼1.21)	0.591
Epinephrine, n (%)	3.86 (1.65, 9)	0.002	3.44 (1.34∼8.83)	0.01
Dopamine, n (%)	3.62 (1.44, 9.08)	0.006	2.52 (0.87∼7.31)	0.089
Norepinephrine, n (%)	3.23 (1.86, 5.61)	<0.001	1.29 (0.58∼2.87)	0.534
Octreotide, n (%)	1.71 (0.81, 3.61)	0.162		
CRRT, n (%)	5.08 (2.81, 9.19)	<0.001	1.7 (0.68∼4.25)	0.254
Ventilator, n (%)	1.43 (0.61, 3.34)	0.407		
Cumulative fluid inputs, Mean ± SD	1 (1,1)	0.008		
Cumulative fluid outputs, Mean ± SD	1 (1,1)	0.196		
Cumulative fluid balance, Mean ± SD	1 (1,1)	0.126		
Urineoutput, Mean ± SD	1 (0.9999, 1)	0.063		
Cumulative fluid positive balance, n (%)	1.79 (1.04, 3.08)	0.034	2.17 (1.18∼4)	0.013

In the multivariate Cox regression analysis before and after PSM, positive fluid balance remained independently associated with 90-day mortality, with E-values of 2.45 and 3.76 for the HRs before and after PSM, respectively. This suggests a certain degree of robustness in the observed association. To further control for confounding by indication and time-dependent confounding, the authors applied the IPTW method. After weighting, the Cox model showed a hazard ratio of 1.51 (95% CI 1.01–2.25, *p* = 0.115) for positive versus negative fluid balance. Although the p-value did not reach conventional statistical significance, the point estimate was consistent with the primary analysis result (HR = 1.54), indicating a persistent positive association.

In landmark analyses (Supplementary Files 4‒5), patients who died within 24 h, 48 h, and 72 h were sequentially excluded. The landmark analysis results showed: at 24 h (HR = 1.62 [1.13–2.32], *p* = 0.009), 48 h (HR = 1.51 [1.05–2.17], *p* = 0.026), and 72 h (HR=1.48 [1.03–2.15], *p* = 0.036). Across these three adjusted analyses, the Kaplan-Meier survival curves were also consistent with the primary finding: positive fluid balance was associated with mortality.

### Nonlinear association between fluid balance and mortality risk

The RCS analysis (Supplementary File 6) revealed a statistically significant overall association between fluid balance and mortality risk, with evidence of a nonlinear relationship (P for non-linearity: < 0.001). Both negative and positive fluid balance were associated with an increased risk of mortality. Although the sample size was limited in the extreme value ranges, introducing a degree of uncertainty in these estimates, this finding suggests the existence of an optimal window for fluid management.

## Discussion

Using the MIMIC-IV v3.1 database, the authors quantified each patient’s cumulative fluid during the initial week of intensive care for a cohort of 1142 adults with AP. Using multiple statistical methods ‒ including univariate and multivariate Cox regression analysis, PSM, K-M survival curves, and subgroup analysis ‒ these findings demonstrate that maintaining positive cumulative fluid balance by Day-7 remains an associated factor for 90-day all-cause mortality in ICU patients with AP. Subgroup analyses and sensitivity analyses validated the consistent association between positive fluid balance and mortality risk in AP patients. Numerous prior studies have examined the relationship between fluid overload and critically ill patients, often demonstrating negative outcomes. This deleterious link implies that a conservative fluid strategy could confer a survival advantage in the critically ill. The present study extends the concept of fluid overload to the AP population, aiming to explore the feasibility of restrictive fluid therapy for AP using a large-scale dataset.

Traditional views hold that early aggressive fluid resuscitation is the cornerstone treatment for AP, aimed at correcting third-space fluid loss and hypovolemia.^19^ However, high-quality randomized controlled trials and meta-analyses in recent years have challenged this notion. The WATERFALL trial, a landmark study, demonstrated that compared to moderate fluid resuscitation, early aggressive fluid resuscitation did not reduce the incidence of moderate-to-severe AP. Instead, it significantly increased the risk of fluid overload and potentially prolonged hospital stays.^20^ This finding was further corroborated by multiple systematic reviews and meta-analyses. Li et al. demonstrated that high-volume resuscitation not only amplifies mortality among individuals with SAP but also elevates fluid-related morbidity across the entire AP spectrum.^21^ Meta-analyses by He et al. and Gad similarly support the conclusion that aggressive fluid resuscitation is associated with higher risks of acute kidney injury and acute respiratory distress syndrome, without improving mortality.^6^^,^^14^ This evidence strongly suggests that for AP patients, particularly those without organ failure at admission, restrictive fluid resuscitation may offer superior safety and efficacy compared to aggressive fluid resuscitation.

By calculating patient fluid volume, the authors define fluid balance as the difference between fluid input and output, which is increasingly recognized as an important prognostic indicator. In AP patients, studies have clearly demonstrated that positive fluid balance correlates with poor outcomes. A multicenter retrospective study by Huang et al. introduced the Fluid Balance Index (FBI), finding that FBI ≥ 145 mL/kg was an independent predictor of in-hospital mortality in critically ill AP patients.^22^ Liu et al. found that positive fluid balance during the 24‒36 hour and 36‒48 hour periods after ICU admission was significantly associated with in-hospital and 30-day mortality in AP patients.^23^ Koop et al. also indicated that a positive 24-hour fluid balance was independently associated with longer hospital stays.^24^ This association extends beyond AP. In sepsis management, Corl et al. found that moderate intravenous fluid resuscitation was associated with lower sepsis-adjusted mortality compared to strategies involving extremely low or extremely high fluid volumes.^25^ Furthermore, Dhondup et al. showed that moving into negative fluid balance during the de-escalation phase of sepsis was linked to a markedly lower 90-day mortality risk, with greater negative balance yielding more pronounced mortality reduction.^26^ Collectively, these studies suggest that closely monitoring and actively avoiding significant positive fluid balance may prove pivotal in enhancing survival across diverse critical-care populations.

Evidence indicates that fluid management should follow the principle of moderation ‒ neither excessive nor insufficient. Weitz's retrospective analysis found that more aggressive fluid therapy in AP patients correlated with disease severity and higher rates of local complications, suggesting potential harm.^27^ Conversely, inadequate fluid resuscitation is equally detrimental. Early research by Brown et al. revealed that patients presenting with blood concentration at admission and failing to achieve a decrease in hematocrit within 24 h all progressed to necrotizing pancreatitis, underscoring the necessity of adequate resuscitation.^28^ de-Madaria's cohort study further refined this perspective, indicating that insufficient fluid administration within the initial 24 h was not associated with poor outcomes, whereas excessive fluid volume was independently linked to risks such as persistent organ failure, suggesting an optimal therapeutic window.^29^ This principle of moderation also applies to other diseases. Balakumar's study found that compared to balanced fluid status, both positive and negative fluid balances were associated with higher 1-year mortality in critically ill patients, depicting a *U*-shaped relationship.^30^ Thus, the goal of fluid therapy should be individualized, goal-directed, and moderate resuscitation, avoiding both extremes.

On Day-1, survivors received significantly more fluid than non-survivors, supporting the established principle that early adequate resuscitation is crucial for correcting hypovolemia and maintaining organ perfusion in acute pancreatitis. However, from Day-3 onwards, fluid balance in survivors showed a decreasing trend, eventually becoming negative, whereas non-survivors remained in a state of persistent positive balance. This pattern suggests that an optimal fluid strategy may be biphasic: an initial liberal resuscitation phase to restore intravascular volume, followed by a de-escalation phase aimed at achieving a negative balance once hemodynamic stability is achieved. The divergence after Day-3 may reflect the resolution of capillary leakage in survivors, while the sustained positive fluid balance in non-survivors likely indicates ongoing inflammation and organ dysfunction.

The present study found that patients in the positive-balance group had higher APS-III and SOFA scores on admission and more frequently received vasoactive drugs and CRRT. These indicators suggest that this cohort may have suffered from more severe systemic inflammatory response and organ dysfunction. Consequently, the observed association between positive fluid balance and mortality may, at least in part, reflect confounding by indication: sicker patients receive more aggressive fluid resuscitation and simultaneously face a higher risk of death because of their underlying disease severity rather than fluid overload per se. This limitation is inherent to the retrospective observational design and cannot be fully eliminated even through statistical multivariable Cox analysis and propensity-score matching. To quantify the potential impact of such confounding, the authors conducted an E-value analysis. This suggests, to some extent, that any unmeasured confounder would need to be exceptionally strong to overturn these findings. In the positive fluid-balance group, the higher rates of vasoactive drugs dependency and CRRT use indicate that these patients already presented with capillary-leak syndrome and multiple-organ dysfunction at admission, such conditions inherently limit fluid responsiveness and predispose to positive balance. In this context, positive fluid balance may serve as a biomarker of disease severity and also a potentially modifiable risk factor, rather than being merely a direct cause of mortality.

A fundamental limitation of this study is the inability to distinguish whether positive fluid balance is a cause of mortality or merely a consequence of disease severity. Patients with more severe capillary leak require larger fluid volumes to maintain perfusion, inevitably leading to a positive balance. In such patients, attempting to achieve a negative balance could theoretically precipitate hypoperfusion and worsen outcomes. Although the authors employed statistical adjustments to reduce confounding, residual confounding by indication likely persists. Therefore, the observed association should not be interpreted as causal.

The authors acknowledge several limitations of this study. First, as a retrospective analysis, despite controlling for known confounders through multivariate regression and PSM, the authors cannot fully exclude the influence of unmeasured or unidentified confounders on outcomes, including the potential for confounding by indication where hemodynamic instability and organ failure drive both increased fluid administration and mortality risk. Additionally, the higher APS-III and SOFA scores in the positive balance group suggest residual confounding from baseline severity. Second, the present study did not explicitly specify fluid types (such as crystalloid or colloid). Different fluid types vary in their volume expansion effects, intravascular retention time, and impact on the internal environment, which may significantly influence fluid balance outcomes.^31^^,^^32^ Finally, all demographic data in this study originated from a single-center ICU, where patients typically present with more severe conditions. The findings may not be directly generalizable to less critically ill AP patients in general wards. Future prospective multicenter studies are needed to validate the relationship between fluid balance and AP mortality risk.

## Conclusion

Within this 1142-patient retrospective AP cohort, cumulative positive fluid balance on Day-7 of admission was independently associated with 90-day all-cause mortality risk. These findings suggest that the relationship between fluid balance and mortality in patients with acute pancreatitis is dynamic. Early adequate fluid resuscitation appears beneficial, whereas a persistent positive balance is associated with increased mortality. This biphasic pattern implies that fluid management should be individualized: initial adequate resuscitation followed by goal-directed de-escalation therapy to achieve negative balance once the patient is stabilized. However, given the observational study design and inherent confounding by indication, this association at least partially reflects the severity of the underlying disease process, rather than a direct causal effect. This hypothesis requires further clinical trials for confirmation and validation.

## Abbreviations

AP, Acute Pancreatitis; SAP, Severe Acute Pancreatitis; MODS, Multiple-Organ-Dysfunction Syndrome; SIRS, Systemic Inflammatory Response Syndrome; ACS, Abdominal Compartment Syndrome; ARDS, Acute Respiratory Distress Syndrome; ICU, Intensive Care Unit; MIMIC-IV v3.1, Medical Information Mart for Intensive Care-IV v3.1; BIDMC, Beth Israel Deaconess Medical Center; CITI, Collaborative Institutional Training Initiative; IRB, Institutional Review Board; ICD, International Classification of Diseases; SD, Standard Deviation; ANOVA, Analysis Of Variance; IQR, Interquartile Range; PSM, Propensity Score Matching; SMDs; Standardized Mean Differences; K-M, Kaplan-Meier; HR, Heart Rate; SBP, Systolic Blood Pressure; DBP, Diastolic Blood Pressure; MAP, Mean Arterial Pressure; RR, Respiratory Rate; SpO_2_, Oxygen Saturation; CHF, Congestive Heart Failure; COPD, Chronic Obstructive Pulmonary Disease; CRRT, Continuous Renal Replacement Therapy; SOFA, Sequential Organ Failure Assessment; APS-III, Acute Physiology Score III; FBI, Fluid Balance Index.

## Ethics approval and consent to participate

The MIMIC-IV database's creation was evaluated by the Institutional Review Board at Beth Israel Deaconess Medical Center, which approved the data-sharing initiative and granted waivers for informed consent. This study adhered to the ethical guidelines outlined in the Declaration of Helsinki. All patient data utilized in this research were de-identified, and therefore, written informed consent was not necessary.

## Consent for publication

Not applicable.

## Data availability

The datasets generated and analyzed during this study are available in the MIMICIV v3.1 database (https://physionet.org/content/mimiciv/3.1/https://physionet.org/content/mimiciv/3.1/).

## Authors' contributions

Jianlei Chen and Hao Li jointly proposed and completed the design of the study. Jianlei Chen, Jinyuan Chi and Hao Li collected and organized all the data, and conducted the statistical analysis. Jianlei Chen wrote the manuscript, which was revised after correction by Hao Li. Hao Li provided administrative support, funding, as well as material and technical support for the study. All authors have read and approved the final manuscript.

## Funding

Not funding.

## Declaration of competing interest

The authors declare no conflicts of interest.

## Appendix Supplementary materials

Image, application 1

## References

[1] Szatmary P., Grammatikopoulos T., Cai W., Huang W., Mukherjee R., Halloran C. (2022). Acute pancreatitis: diagnosis and treatment. Drugs.

[2] Szatmary P., Grammatikopoulos T., Cai W., Huang W., Mukherjee R., Halloran C. (2022). Acute pancreatitis: diagnosis and treatment. Drugs.

[3] Garg P.K., Singh V.P. (2019). Organ failure due to systemic injury in acute pancreatitis. Gastroenterology.

[4] Nolan J.P., Sandroni C., Böttiger B.W., Cariou A., Cronberg T., Friberg H. (2021). European resuscitation council and European society of intensive care medicine guidelines 2021: post-resuscitation care. Intensive Care Med.

[5] Trikudanathan G., Yazici C., Evans A.P., Forsmark C.E. (2024). Diagnosis and management of acute pancreatitis. Gastroenterology.

[6] He K., Gao L., Yang Z., Zhang Y., Hua T., Hu W. (2023). Aggressive versus controlled fluid resuscitation in acute pancreatitis: a systematic review and meta-analysis of randomized controlled t.rials. Chin Med J.

[7] Harrell B.R., Miller S. (2017). Abdominal compartment syndrome as a complication of fluid resuscitation. Nurs Clin North Am.

[8] van Mourik N., Metske H.A., Hofstra J.J., Binnekade J.M., Geerts B.F., Schultz M.J. (2019). Cumulative fluid balance predicts mortality and increases time on mechanical ventilation in ARDS patients: an observational cohort study. PLoS One.

[9] Hofer D.M., Ruzzante L., Waskowski J., Messmer A.S., Pfortmueller C.A. (2024). Influence of fluid accumulation on major adverse kidney events in critically ill patients: an observational cohort study. Ann Intensive Care.

[10] Messmer A.S., Zingg C., Müller M., Gerber J.L., Schefold J.C., Pfortmueller C.A. (2020). Fluid overload and mortality in adult critical care patients: a systematic review and meta-analysis of observational studies. Crit Care Med.

[11] Salahuddin N., Sammani M., Hamdan A., Joseph M., Al-Nemary Y., Alquaiz R. (2017). Fluid overload is an independent risk factor for acute kidney injury in critically ill patients: results of a cohort study. BMC Nephrol.

[12] Acheampong A., Vincent J.L. (2015). A positive fluid balance is an independent prognostic factor in patients with sepsis. Crit Care.

[13] Rosenberg A.L., Dechert R.E., Park P.K., Bartlett R.H. (2009). Review of a large clinical series: association of cumulative fluid balance on outcome in acute lung injury: a retrospective review of the ARDSnet tidal volume study cohort. J Intensive Care Med.

[14] Gad M.M., Simons-Linares C.R. (2020). Is aggressive intravenous fluid resuscitation beneficial in acute pancreatitis? A meta-analysis of randomized control trials and cohort studies. World J Gastroenterol.

[15] Johnson A.E.W., Bulgarelli L., Shen L., Gayles A., Shammout A., Horng S. (2023). MIMIC-IV, a freely accessible electronic health record dataset. Sci Data.

[16] Renaudier M., Lascarrou J.B., Chelly J., Lesieur O., Bourenne J., Jaubert P. (2025). Fluid balance and outcome in cardiac arrest patients admitted to intensive care unit. Crit Care.

[17] Kane L.T., Fang T., Galetta M.S., Goyal D.K.C., Nicholson K.J., Kepler C.K. (2020). Propensity score matching: a statistical method. Clin Spine Surg.

[18] VanderWeele T.J., Ding P. (2017). Sensitivity analysis in observational research: introducing the E-value. Ann Intern Med.

[19] Aggarwal A., Manrai M., Kochhar R. (2014). Fluid resuscitation in acute pancreatitis. World J Gastroenterol.

[20] de-Madaria E., Buxbaum J.L., Maisonneuve P., García de Paredes A.G., Zapater P., Guilabert L. (2022). Aggressive or moderate fluid resuscitation in acute pancreatitis. N Engl J Med.

[21] Li X.W., Wang C.H., Dai J.W., Tsao S.H., Wang P.H., Tai C.C. (2023). Comparison of clinical outcomes between aggressive and non-aggressive intravenous hydration for acute pancreatitis: a systematic review and meta-analysis. Crit Care.

[22] Huang X., Xu Z., Liu S., Liu X., Lin L., Pan M. (2025). Association of fluid balance index with in-hospital mortality in critically ill patients with acute pancreatitis: a multicenter retrospective cohort study. World J Emerg Med.

[23] Liu L., Wang C., Luo T., Li L. (2020). Effects of fluid resuscitation on the occurrence of organ failure and mortality in patients with acute pancreatitis. Pancreas.

[24] Koop A.H., Stancampiano F.F., Jackson J., Henry A., Horsley-Silva J., Pannala R. (2018). Association of total fluid intake and output with duration of hospital stay in patients with acute pancreatitis. Gastroenterol Res Pract.

[25] Corl K.A., Levy M.M., Holder A.L., Douglas I.S., Linde-Zwirble W.T., Alam A. (2024). Moderate IV fluid resuscitation is associated with decreased sepsis mortality. Crit Care Med.

[26] Dhondup T., Tien J.C., Marquez A., Kennedy C.C., Gajic O., Kashani K.B. (2020). Association of negative fluid balance during the de-escalation phase of sepsis management with mortality: a cohort study. J Crit Care.

[27] Weitz G., Woitalla J., Wellhöner P., Schmidt K., Büning J., Fellermann K. (2014). Detrimental effect of high-volume fluid administration in acute pancreatitis: a retrospective analysis of 391 patients. Pancreatology.

[28] Brown A., Baillargeon J.D., Hughes M.D., Banks P.A. (2002). Can fluid resuscitation prevent pancreatic necrosis in severe acute pancreatitis?. Pancreatology.

[29] de-Madaria E., Soler-Sala G., Sánchez-Payá J., Lopez-Font I., Martínez J., Gómez-Escolar L. (2011). Influence of fluid therapy on the prognosis of acute pancreatitis: a prospective cohort study. Am J Gastroenterol.

[30] Balakumar V., Murugan R., Sileanu F.E., Palevsky P., Clermont G., Kellum J.A. (2017). Both positive and negative fluid balance may be associated with reduced long-term survival in the critically ill. Crit Care Med.

[31] Dawson A., Karunakaran M., Sharma Z.D., Ullah S., Barreto S.G. (2023). Fluid resuscitation in the early management of acute pancreatitis: evidence from a systematic review and meta-analysis. HPB.

[32] Tseng C.H., Chen T.T., Wu M.Y., Chan M.C., Shih M.C., Tu Y.K. (2020). Resuscitation fluid types in sepsis, surgical, and trauma patients: a systematic review and sequential network meta-analyses. Crit Care.

