# Supplementary material for: Correlation between early cumulative fluid balance and 90-day all-cause mortality in patients with acute pancreatitis: a retrospective cohort study
Source: Clinics (Sao Paulo). 2026 Jun 2;81:101019. doi: 10.1016/j.clinsp.2026.101019 (PMC13251699; doi:10.1016/j.clinsp.2026.101019)

CLINICS-D-25-01822_Supplementary Material

**Supplementary File 1** Comparison of matched pairs after PSM.

| **Variables** | **Total**  **(n = 346)** | **Negative balance**  **(n = 173)** | **Positive balance**  **(n = 173)** | **p** |
| --- | --- | --- | --- | --- |
| Gender, n (%) |  |  |  | 0.745 |
| Female | 153 (44.2) | 75 (43.4) | 78 (45.1) |  |
| Male | 193 (55.8) | 98 (56.6) | 95 (54.9) |  |
| Age, n (%) |  |  |  | 0.59 |
| < 60 | 181 (52.3) | 88 (50.9) | 93 (53.8) |  |
| ≥ 60 | 165 (47.7) | 85 (49.1) | 80 (46.2) |  |
| Race, n (%) |  |  |  | 0.72 |
| White | 202 (58.4) | 104 (60.1) | 98 (56.6) |  |
| Black | 35 (10.1) | 18 (10.4) | 17 (9.8) |  |
| Others | 109 (31.5) | 51 (29.5) | 58 (33.5) |  |
| HR, Mean ± SD | 96.4 ± 21.1 | 95.9 ± 21.7 | 97.0 ± 20.5 | 0.638 |
| SBP, Mean ± SD | 126.1 ± 26.1 | 127.5 ± 27.3 | 124.8 ± 24.9 | 0.341 |
| DBP, Mean ± SD | 70.1 ± 17.7 | 69.9 ± 17.6 | 70.3 ± 17.8 | 0.83 |
| MAP, Mean ± SD | 84.0 ± 19.0 | 84.1 ± 19.3 | 83.9 ± 18.7 | 0.918 |
| RR, Mean ± SD | 21.2 ± 6.3 | 21.3 ± 6.4 | 21.0 ± 6.2 | 0.665 |
| Temperature, Mean ± SD | 36.9 ± 0.9 | 37.0 ± 0.8 | 36.9 ± 0.9 | 0.149 |
| SpO_2_, Mean ± SD | 96.0 ± 4.5 | 96.0 ± 4.8 | 96.1 ± 4.1 | 0.838 |
| CHF, n (%) |  |  |  | 0.814 |
| No | 244 (70.5) | 123 (71.1) | 121 (69.9) |  |
| Yes | 102 (29.5) | 50 (28.9) | 52 (30.1) |  |
| COPD, n (%) |  |  |  | 0.278 |
| No | 251 (72.5) | 121 (69.9) | 130 (75.1) |  |
| Yes | 95 (27.5) | 52 (30.1) | 43 (24.9) |  |
| Diabetes, n (%) |  |  |  | 0.906 |
| No | 243 (70.2) | 122 (70.5) | 121 (69.9) |  |
| Yes | 103 (29.8) | 51 (29.5) | 52 (30.1) |  |
| Renal disease, n (%) |  |  |  | 0.406 |
| No | 282 (81.5) | 144 (83.2) | 138 (79.8) |  |
| Yes | 64 (18.5) | 29 (16.8) | 35 (20.2) |  |
| Cancer, n (%) |  |  |  | 0.47 |
| No | 312 (90.2) | 154 (89) | 158 (91.3) |  |
| Yes | 34 ( 9.8) | 19 (11) | 15 (8.7) |  |
| Liver disease, n (%) |  |  |  | 0.873 |
| No | 301 (87.0) | 151 (87.3) | 150 (86.7) |  |
| Yes | 45 (13.0) | 22 (12.7) | 23 (13.3) |  |
| Sepsis, n (%) |  |  |  | 0.245 |
| No | 77 (22.3) | 34 (19.7) | 43 (24.9) |  |
| Yes | 269 (77.7) | 139 (80.3) | 130 (75.1) |  |
| Charlson average score, Median (IQR) | 4.0 (2.0, 6.0) | 4.0 (2.0, 6.0) | 4.0 (2.0, 6.0) | 0.83 |
| APSIII average score, Mean ± SD | 55.1 ± 23.8 | 51.9 ± 21.9 | 58.2 ± 25.1 | **0.012** |
| SOFA average score, Median (IQR) | 5.9 ± 3.1 | 5.4 ± 2.8 | 6.5 ± 3.3 | **0.001** |
| Epinephrine, n (%) |  |  |  | 0.396 |
| No | 333 (96.2) | 168 (97.1) | 165 (95.4) |  |
| Yes | 13 (3.8) | 5 (2.9) | 8 (4.6) |  |
| Dopamine, n (%) |  |  |  | 1 |
| No | 336 (97.1) | 168 (97.1) | 168 (97.1) |  |
| Yes | 10 (2.9) | 5 (2.9) | 5 (2.9) |  |
| Norepinephrine, n (%) |  |  |  | **0.016** |
| No | 206 (59.5) | 114 (65.9) | 92 (53.2) |  |
| Yes | 140 (40.5) | 59 (34.1) | 81 (46.8) |  |
| Octreotide, n (%) |  |  |  | 0.458 |
| No | 314 (90.8) | 155 (89.6) | 159 (91.9) |  |
| Yes | 32 (9.2) | 18 (10.4) | 14 (8.1) |  |
| CRRT, n (%) |  |  |  | 0.081 |
| No | 317 (91.6) | 163 (94.2) | 154 (89) |  |
| Yes | 29 (8.4) | 10 (5.8) | 19 (11) |  |
| Ventilator, n (%) |  |  |  | 0.76 |
| No | 50 (14.5) | 24 (13.9) | 26 (15) |  |
| Yes | 296 (85.5) | 149 (86.1) | 147 (85) |  |
| 90-d mortality, n (%) |  |  |  | **0.041** |
| No | 290 (83.8) | 152 (87.9) | 138 (79.8) |  |
| Yes | 56 (16.2) | 21 (12.1) | 35 (20.2) |  |

**Supplementary File 2** Standardized mean differences after propensity score matching.

| **Variable** | **SMD** | |
| --- | --- | --- |
|  | **Before matching** | **After matching** |
| Male, n (%) | 0.043 | 0.035 |
| Age ≥ 60, n (%) | 0.071 | 0.058 |
| Race, n (%) | 0.04 | 0.087 |
| White |  |  |
| Black |  |  |
| Others |  |  |
| HR, Mean ± SD | 0.069 | 0.051 |
| SBP, Mean ± SD | 0.291 | 0.102 |
| DBP, Mean ± SD | 0.315 | 0.023 |
| MAP, Mean ± SD | 0.274 | 0.011 |
| RR, Mean ± SD | 0.096 | 0.047 |
| Temperature, Mean ± SD | 0.009 | 0.156 |
| SpO_2_, Mean ± SD | 0.092 | 0.022 |
| CHF, n (%) | 0.112 | 0.025 |
| COPD, n (%) | 0.104 | 0.117 |
| Diabetes, n (%) | 0.071 | 0.013 |
| Renal disease, n (%) | 0.137 | 0.089 |
| Cancer, n (%) | 0.127 | 0.078 |
| Liver disease, n (%) | 0.21 | 0.017 |
| Sepsis, n (%) | 0.527 | 0.125 |
| Charlson average score, Median (IQR) | 0.246 | 0.03 |
| APSIII average score, Mean ± SD | 0.502 | 0.271 |
| SOFA average score, Median (IQR) | 0.86 | 0.352 |
| Epinephrine, n (%) | 0.121 | 0.091 |
| Dopamine, n (%) | 0.071 | <0.001 |
| Norepinephrine, n (%) | 0.48 | 0.261 |
| Octreotide, n (%) | 0.174 | 0.08 |
| CRRT, n (%) | 0.186 | 0.189 |
| Ventilator, n (%) | 0.304 | 0.033 |

**Supplementary File 3** Changes in standardized mean differences of covariates before and after PSM.


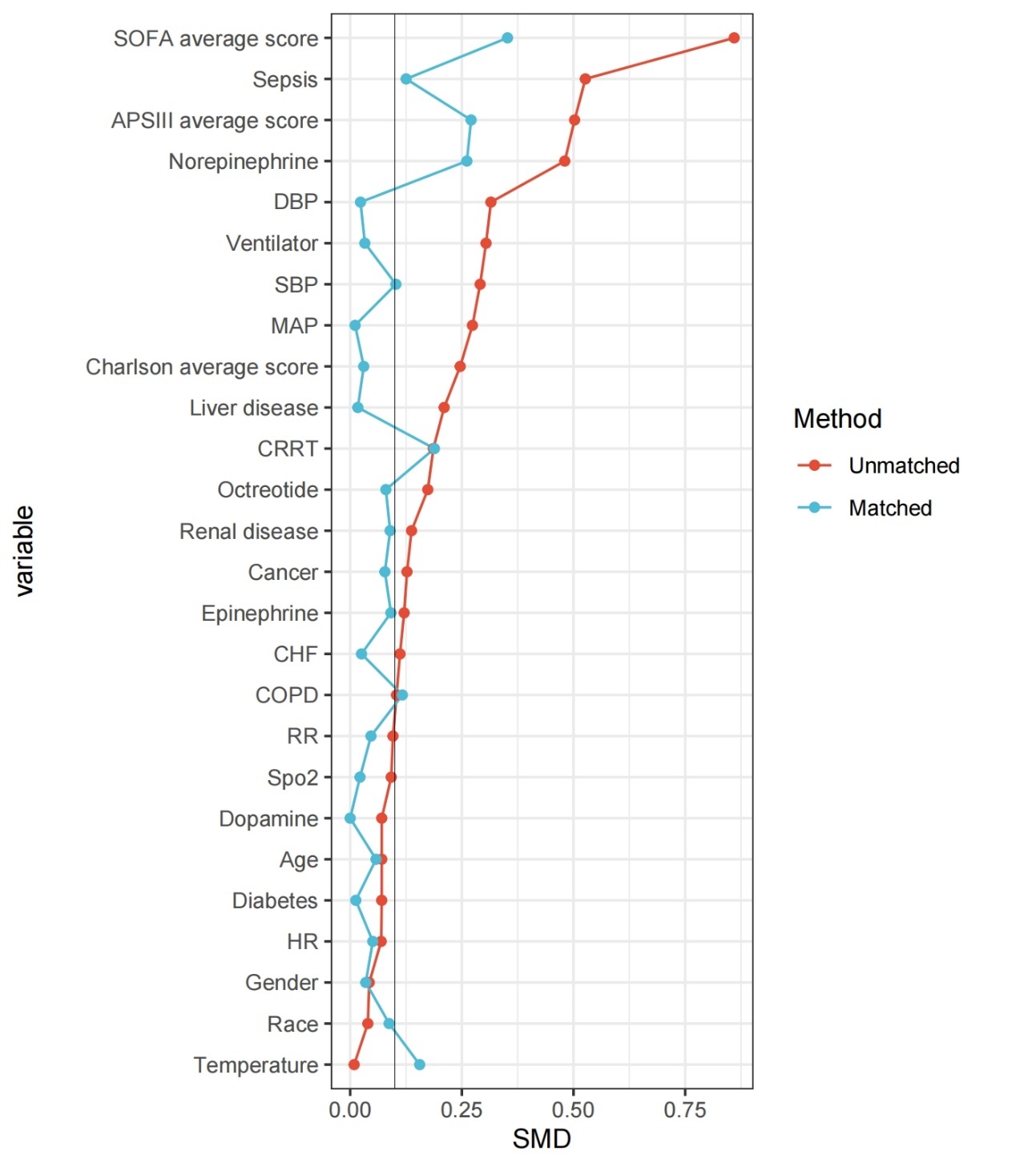


**Supplementary File 4** Landmark analysis of the association between positive fluid balance and 90-Day mortality, excluding patients who died within 24h, 48h, and 72h.

| **Item** | **Group** | **Number of patients** | **HR (95% CI)** | **p-value** |
| --- | --- | --- | --- | --- |
| at 24h | Negative balance | 300 | 1(Ref) |  |
|  | Positive balance | 822 | 1.62 (1.13~2.32) | **0.009** |
| at 48h | Negative balance | 300 | 1(Ref) |  |
|  | Positive balance | 806 | 1.51 (1.05~2.17) | **0.026** |
| at 72h | Negative balance | 299 | 1(Ref) |  |
|  | Positive balance | 799 | 1.48 (1.03~2.15) | **0.036** |

**Supplementary File 5** Landmark analysis of K-M survival curves: (a) Excluding patients who died within 24h, (b) Excluding patients who died within 48h, and (c) Excluding patients who died within 72h.


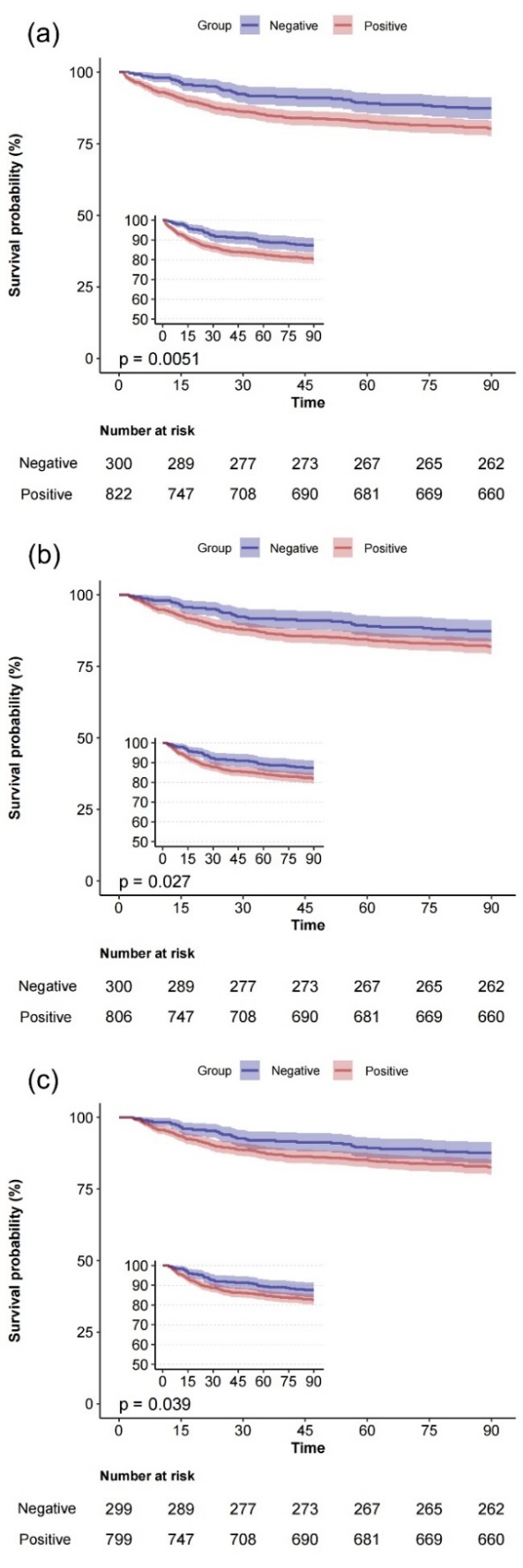


**Supplementary File 6** Restricted cubic spline analysis of the nonlinear association between cumulative fluid balance and 90-day mortality risk.


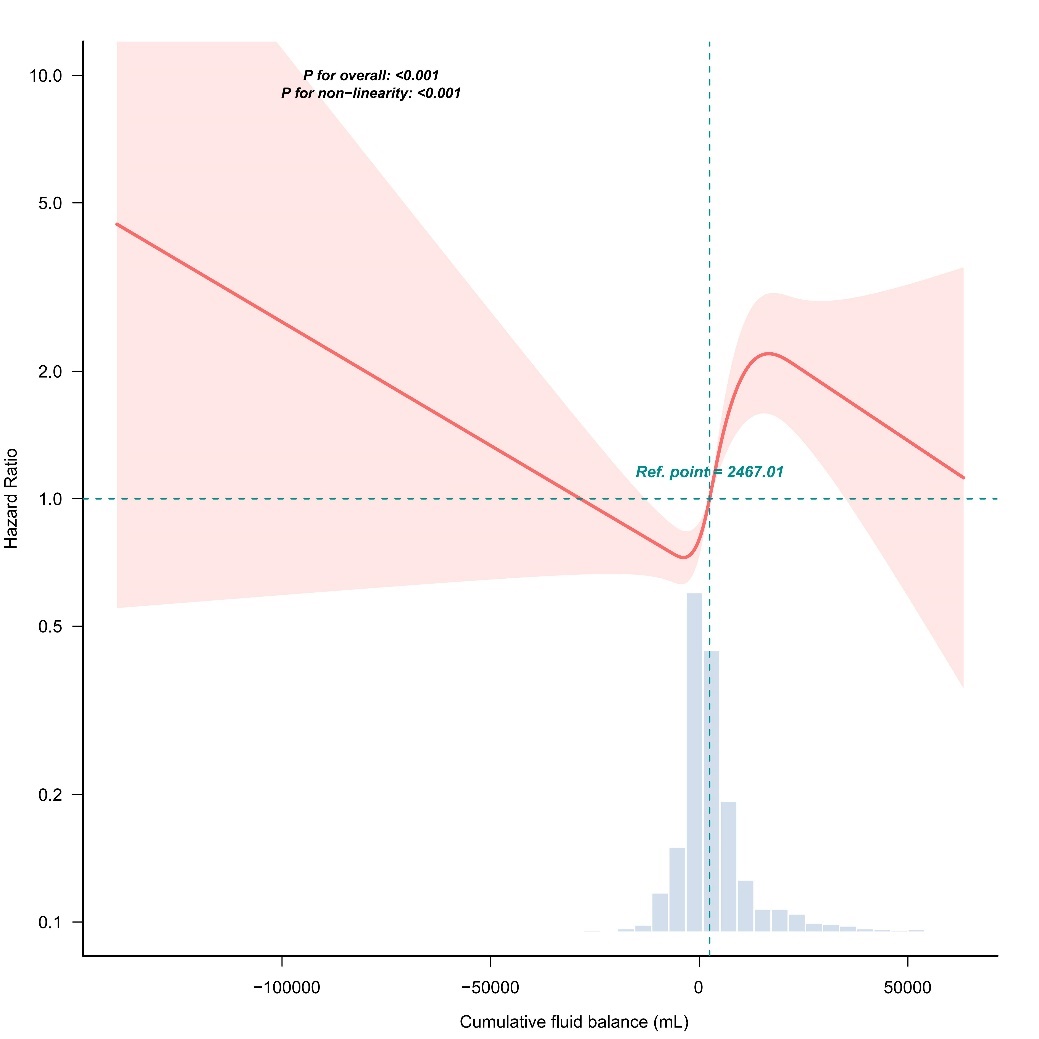

Supplement: Supplementary file 1 [file mmc1.docx]
